# Dementia, Preclinical Studies in Neurodegeneration and its Potential for Translational Medicine in South America

**DOI:** 10.3389/fnagi.2016.00304

**Published:** 2016-12-20

**Authors:** Gloria Patricia Cardona-Gómez, Francisco Lopera

**Affiliations:** ^1^Cellular and Molecular Neurobiology Area, Neuroscience Group of Antioquia, Faculty of Medicine, Sede de Investigación Universitaria (SIU), University of AntioquiaMedellin, Colombia; ^2^Clinical Neuroscience Area, Neuroscience Group of Antioquia, Faculty of Medicine, Sede de Investigación Universitaria (SIU), University of AntioquiaMedellin, Colombia

**Keywords:** neurodegeneration, preclinical studies, pharmacological therapy, gene therapy, translational medicine

## Abstract

Latin-American people with dementia will increase to an astounding 368% in 2050, higher than USA and Europe. In addition, to sporadic dementia type like Alzheimer, and vascular dementia (VaD) progression after Cerebrovascular disease is also found. These incidences are increased in Colombia by specific populations affected with pure Neurodegenerative and VaDs like Autosomical Dominant familial Alzheimer’s disease (AD) and Cerebral Autosomal-Dominant Arteriopathy with Subcortical Infarcts and Leukoencephalopathy (CADASIL). In spite of the enormous human effort with and economical effort and investment costs, neither sporadic nor genetic kinds of dementia progression have been prevented or blocked yet. Currently, there exist several animal models that partially solve the understanding of the neurodegenerative etiopathogenesis and its treatment. However, when the potential therapies are translated to humans, those do not work or present a limited action. Main difficulties are the diverse comorbility associated to the cause and/or several affected brain regions, reducing the efficacy of some therapies which are limited to a tissue-specific action or modulating a kind of neurotransmission. Global investigation suggests that a general prevention could be achieved with the improvement in the quality of lifestyle, including healthy diet, physical and mental activity, and avoiding mechanical or chemical pro-inflammatory events in an early stage in the most of non-communicable diseases. In this review article, we present some molecular targets and preclinical studies in animal models to propose strategies that could be useful in a future translation to prevent or block neurodegeneration: one is gene therapy; silencing pathogenic genes in critical brain areas where excitotoxicity arise and spread. Another is to take advantage of the natural source and its wide biodiversity of natural products that are capable of identifying, by the blocking and prevention of neurodegeneration. On the other side, the casuistic of pure dementias in the Latin-American region gives an exceptional opportunity to understand the pathogenesis in these human populations. Further, this is in support of the basic and clinical researchers working on an interaction for a better understanding and medical care of mixed dementias, which have more complex factors than pure ones. However, to promote the translation of any therapeutical alternative is necessary to clarify the normative and the protocols for developing clinical trials with original candidates or work upon strategies proposed from South-American countries.

## Dementia Epidemiology: Chronic and Acute Brain Injury

Mental and neurological disorders are prevalent worldwide. Traditional epidemiological studies represent and indicate only mortality levels, and seriously underestimate the social impact of neurodegeneration; because it does not consider disability levels. The latest report on the global burden of disease shows that neurodegenerative disorders generate 1% of mortality; out of the 11.2% of disability reported worldwide. Life expectancy increase in industrialized and developing countries leads to neurological disorders and are becoming a major public health problem that could reach pandemic proportions in the coming years. It is anticipated that by 2020 the levels of disability due to neurological diseases will be above 14.7% (WHO, 2016).

Dementia (Alzheimer’s disease (AD) type, vascular dementia (VaD), and other dementias) is a disease that affects more than 26 million people worldwide. Latin-American people with dementia will increase in a 368% in 2050, higher than USA and Europe (Informe ADI/Bupa, 2013; WHO, 2016). As a result of these statistical projections, clear and detailed programs are needed for capacity building in Latin-America to handle the volumes due to increased casuistic (Gonzalez et al., 2014). The prevalence of Dementia is South America exists in multiple variations due to Population, Age Structure, Genetics, life style of people who are older than 65 years. In South America there exists a big variation in the dementia prevalence, according to population indicators like population age structure, Genetics and Lifestyle of people older than 65 years, Whereas in Argentina (11.5%) and Venezuela (10.3%) present a higher prevalence of dementia, Cuba (8.2%), Brazil (5.3%), Perú (6.7%), Chile (4.3%) present an intermediate prevalence, although some lower prevalence exists in Colombia (1.8%), Uruguay (0.5%) and some cities in Brazil (1%–2%) and in rural Perú (<1%; Kalaria et al., 2008). In Colombia, people with dementia and other cognitive disorders are increasing due to the closed relationship with public health problems, such as metabolic disorders like diabetes, hypertension, cerebrovascular disease and accidents. In the Antioquia department in Colombia, the situation is more critical, due to the high incidence of familial AD. On an average more than 1000 individuals will develop the disease before reaching old age, apart from other dementias like Cerebral Autosomal-Dominant Arteriopathy with Subcortical Infarcts and Leukoencephalopathy (CADASIL) with more than 80 affected individuals per 1000. Also, an analog situation occurs in Venezuela, with an estimated Huntington’s disease prevalence of 720 per 100,000 habitants in Maracaibo (Al-Jader et al., 2001).

AD is the most common cause of dementia in people over 65 years of age, including more than 20 million people are affected by sporadic AD worldwide. AD is characterized by aggregated protein that forms amyloid plaques and neurofibrillary tangles (NTFs), causing brain atrophy and cognitive impairment progression leading to dementia. Protein’s aggregates are a common feature of several neurodegenerative diseases, however, some cellular accumulated proteins in the senescent brains, do not cause cognitive disorders, suggesting that, this might be the result of normal aging processes (Przedborski et al., 2003). AD has an important vascular component (Muresanu et al., 2014), and recently it has been considered that early detection of proto-fibrillar amyloid in young Familiar AD (FAD) patients by PET (Reiman et al., 2012), could be an important biomarker for the prevention of vascular deterioration, as the common cause of VaD. Nevertheless, aggregation of the tau protein is an exception. Studies that evaluate the brains of AD patients have found that the number of NTFs correlates with dementia more accurately than the amyloid plaques (Arriagada et al., 1992) in the later stage. This evidence generates great interest for therapeutic intervention in AD and other tauopathies (Ballatore et al., 2007), by chronic or acute injuries.

VaD is the second most common type of dementia, small-artery associated to hypertensive disease is a common cause around the world (73%), but presenting a low prevalence from 0.6% to 2.1% in people older to 65 years in developing countries, maybe due to poor early diagnosis and a subdiagnosis as mix dementia, which has a prevalence of 10% in Latin-American (Kalaria et al., 2008). Mix dementia combines more than one type of dementia presenting deposits associated to AD and vascular affection, which may coexist with Lewy bodies, typical of Parkinson’s dementia (Alzheimer’s association, 2016). Therefore, it is difficult to determine its prevalence, based upon post-morten determination. However it is closely correlated with the increased age and is also higher in the senior patients in age above >85 year old. In addition these symptoms depend upon the affected brain regions, but are also commonly diagnosed as Alzheimer’s patients (94%), coexisting with vascular disease and Lewy bodies and other such nonspecific treatments have also been included (Alzheimer’s association, 2016).

On the other hand, developing countries have a higher level of exposure to cerebrovascular risk factors, such as hypertension, smoking, obesity and diabetes, than in developed countries (Rizzi et al., 2014); which increase the probability for suffering an ischemic stroke, which is a major risk factor for developing VaD and AD (Vijayan and Reddy, 2016). Cerebral ischemia is a type of stroke characterized by transient or permanent decreas in blood flow due to thrombotic occlusion or embolic in one or more cerebral arteries. This results in the deprivation of trophic factors, metabolic substrates and the activation of cell death pathways (Roberts et al., 2013). Depending on the impact of ischemia (duration of reduced blood flow and infarct location), this disease can cause various clinical manifestations. Common afflictions are Paralysis or Hemiplegia, Aphasia, problems associated with processes of memory and learning and similar other effects (Kemp and McKernan, 2002). Stroke is responsible for more than five million deaths each year worldwide, making it the second highest leading cause of death and a major cause of permanent mental and physical disability (Wen et al., 2004; Zheng et al., 2010). Accordingly to the World Health Organization (WHO) Argentina, Chile, Brazil and Uruguay present 6–11 per 1000 affected persons in comparison with 5–6 per 1000 habitants in South-America; being higher in Brazil (11 per 1000) and lower in rural Bolivia (1 per 1000; Lavados et al., 2007). Cerebrovascular accident is the second cause of death in people over 45 years of age in Colombia (Ministry of health and social protection, Colombia 2015). It is estimated that one in six people may suffer at least one episode of cerebral ischemia in life and only one in three survive as patients that too in a state of physical dependence, such cases could also develop dementia (Durukan and Tatlisumak, 2007). Stroke remains one of the cause of high social and individual cost of employee disability, leading to high spending on health and generating a negative impact on the economy of the region itself, therefore cerebral ischemia is a priority within the current scientific research.

Several studies have demonstrated presence of senile plaques (βA) and amyloid precursor protein (APP) in close proximity to the ischemic focus (Ikeda et al., 2000; Shi et al., 2006), suggesting a degree of convergence in the neuropathogenesis of cerebral ischemia and AD (Zekry et al., 2003; Snyder et al., 2015; Kalaria et al., 2016; Nelson et al., 2016). In stroke pathophysiology, changes in the phosphorylation pattern of the Tau protein during and after the ischemic event are observed (Wen et al., 2004; Gutiérrez-Vargas et al., 2016). After infarction, the rapid dephosphorylation of Tau occurs, and after blood reperfusion, there is evidence of slow but steady hyperphosphorylation, which causes an accumulation of the tau protein, resulting in long-term brain damage (Wen et al., 2004). Additionally, cerebral ischemia contributes to the development of cognitive decline and dementia, which is induced by a sedentary lifestyle, unhealthy eating habits, diabetes and other metabolic diseases (Vijayan and Reddy, 2016). Epidemiological studies have also shown that the prevalence of cognitive impairment in ischemic stroke patients is nine-fold higher and controls at 3 months and 4–12 times higher and controls 4 years after stroke, as a result three out of four patients are probable to develop dementia (Wen et al., 2004), predominantly AD-type (Pluta, 2004).

However, with respect to the cognitive impairment definition, it is necessary to clarify that it is a clinical sign related to cerebrovascular disease, but it has not been precisely quantified as a vascular cognitive impairment (VCI) incidence (Chui, 2005). Hence a vascular cognitive disorder spectrum exists between VCI to VaD (Román et al., 2004). In general, VaD is considered a deficit of new learning and memory issues more than the pattern of motor slowing and executive deficit. Some researchers suggest separated definitions, but epidemiological studies suggest a continuum of AD’s disease and vascular brain disease. Also, microvascular disease is correlated with the advanced age, but not completely correlated with cognitive impairment (Selnes and Vinters, 2006). Therefore, a better understanding between vascular disease to cognitive decline and dementia risk is necessary. Maybe an early treatment of vascular affection may reduce the probability to develop dementia (Stephan et al., 2009). Also, it is necessary to unify some criteria for defining differential treatments, depending upon the ethiopathogenesis of VCI, because most of the risk factors are common to AD (Venkat et al., 2015).

Recent scientific advances support that vascular risk factors linked to cerebrovascular disease and stroke in the elderly, significantly increase the risk of developing AD. These include atherosclerosis, atrial fibrillation, coronary artery disease, hypertension and diabetes mellitus. Moreover, review of various autopsy series shows that 60%–90% of AD cases exhibit variable cerebrovascular pathology. Although some vascular lesions such as cerebral amyloid angiopathy, endothelial degeneration, and periventricular white matter lesions are evident in most cases of AD and a third would exhibit cerebral infarction (Kalaria, 2000; Snyder et al., 2015; Kalaria et al., 2016). The development and progression of both AD neuropathology and cerebrovascular lesions in the central nervous system are characterized by excitotoxicity. Activation of glial cells and upregulation of inflammatory mediators (Sandu et al., 2015), in a particular temporal, causes the disease progression converging in neurovascular affection, loss of connectivity and dementia. Indeed, maybe an anti-spreading of excitotoxicity and anti-inflammatory approaches have proven to be beneficial in the prevention and treatment of a wider spectrum of vascular cognitive impairment and dementia (VCID).

## Current Therapeutical Approach: An Invitation to Change the Lifestyle

**Figure 1:**
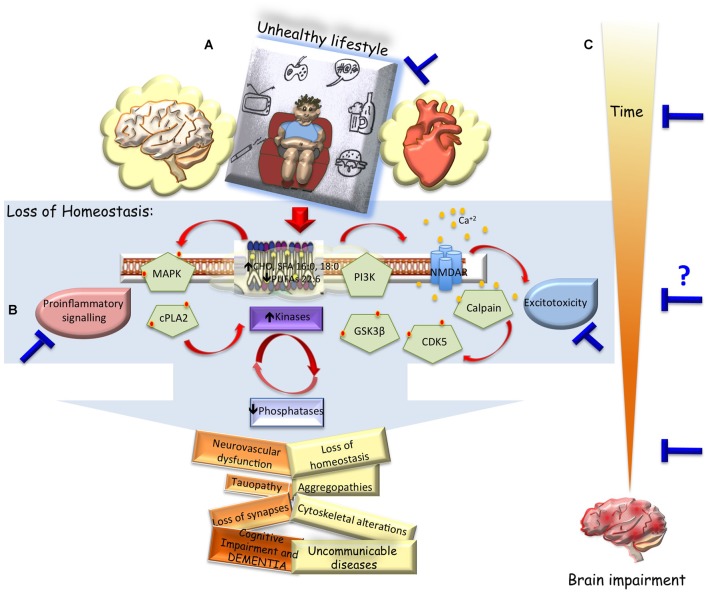
**Unhealthy lifestyle vs. dementia and therapeutical approach. (A)** Hypothetical scheme representing how an unhealthy lifestyle could change the cell environment, triggering imbalanced cell signaling and lossing intrinsic regulation (altered saturated/unsaturated phospholipid composition, spreading of excitotoxicity, kinases/phosphates disbalance, pro-inflammatory response). Which could generate progressive affection of systemic functions (v.gr. metabolic disorders, diabetes, hypertension, atherosclerosis), in general; and cerebral dysfunction in particular, inducing loss of connectivity, vascular cogntivie impairment and dementia. **(B)** Convergent disbalance events in the brain after acute or chronic injury, which could be blocked by change of habits, or pharmacological and gene therapies in pivotal events (blue bars). **(C)** Being the main difficulty to define the inclusion criteria and the intervention time for translational approach, but highlighting the diverse therapeutical windows opportunities and phases of prevention of brain impairment.

Currently, exist several animal models that partially solve the understanding of the neurodegenerative etiopathogenesis and its treatment. However, when the potential therapies are translated to humans it did not work or presented only a limited action. Main difficulties are the diverse comorbility associated to the cause and/or multiple affected brain regions. This reduces the efficacy of some therapies that are limited to a tissue-specific action or neurotransmission modulating types. Global investigation suggests that a general prevention could be achieved with the improvement in the quality of lifestyle, including healthy diet, physical and mental activity, and avoiding mechanical or chemical pro-inflammatory events in an early stage of the most of non-communicable diseases (NIH, 2016; WHO, 2016). Sedentary lifestyle, high calories ingest, little or nil physical and mental activity, lead to metabolic disorders such as, hyperglycemia, dyslipidemia, hyperthension, diabetes, tissue pro-immflamatory environment. These unhealthy circumstances are a common cause of neurodegeneration due to loss of kinases/phosphatases balance and reduces brain plasticity, independently of the etiopathogenesis, which leads to Sickness, Aging and high probability to develop VCID (Figure 1A). Therefore, the best prevention of brain diseases is to promote a healthy lifestyle, including balanced nutrition, exercise, mental activity and social relationships apart from providing motivation and integrated health to the people (Figure 1B).

However, once it has started the disease must be defined how and when to treat, which continues to being a big challenge. Important progress has been made in understanding the neurodegeneration progress in AD and its treatment, for example; some medications have been approved by the FDA. But therapeutical approaches provide only a modest relief of cognitive symptoms, including disturbances in memory and perception (Bassil and Grossberg, 2009; Neugroschl and Sano, 2009); ameliorating symptoms but do not alter the course of disease progression and have even shown some undesired side effects (Soreq and Seidman, 2001; Bassil and Grossberg, 2009; Mimica and Presecki, 2009). Currently, available drugs for AD’s treatment are glutamate modulators and acetyl-cholinesterase (AChE) inhibitors with just palliative outreach. Glutamate modulators continue being studied for improving its action (Danysz and Parsons, 2012), because they have poor effect in severe AD patients and carry several side effects. AChE has been detected in senile plaques (Gomez-Ramos et al., 1992), and its inhibition reduces the metabolic degradation of ACh slowing the progression of cognitive dysfunction. In consequence, AChE inhibitors are indicated in the early stage of the disease, delaying the memory and attention deterioration. This treatment is combined with other drugs for associated AD symptons, such as depression, agitation, sleep disturbances, or later complications like sphincter incontinence, urinary infections, ulcers and thrombophlebitis caused by immobility. However, many of these drugs have several side effects, such as diarrhea, dizziness, loss of appetite, muscle cramps, nausea, fatigue, insomnia, vomit, weight loss and hepatotoxicity, reducing the use of those medicaments.

Other research have applied immunotherapy for AD treatment, for example it has been developed that the AN-1792 vaccine (Tabira et al., 2011), which is a synthetic form of βA protein, that could stimulate the immune system by eliminating βA plaques and preventing emergence of new ones. But adverse effects like development of meningoencephalitis have been identified, resulting in non-approval of the treatment (Delrieu et al., 2012). Currently, there are several immunotherapy clinical trials in phase II and III, which evaluate new strategies for blocking βA, or at least for slowing the disease progression (Mangialasche et al., 2010). For example, the placebo-controlled first prevention trial of crenezumab immunotherapy developed by Genentech, Banner and our research group “GNle of A”, is being evaluated with high risk members from families carrying the E280A-PS1 AD mutation, before onset of mild cognitive impairment (MCI) stage (Neurology, 2012). However, new therapeutical agents capable of blocking cognitive impairment progress in AD must be identified. At the same time it is necessary to determine the critical event that triggers the disease’s development, and for trying to offer not only a slower or a palliative approach, but ideally to offer a preventive or curative treatment without side effects on the patient’s health. Currently these are not available on the market.

On the other hand, today, according to the National Institute of Infarction in USA, the only therapy approved by the FDA for using in the first 4.5 h post-ischemic stroke is the thrombolytic therapy with recombinant tissue plasmilógeno (tPA; Hanger et al., 2007). This fibrinolytic process is a cost-effective treatment: which does not increase process costs and is efficient because it reduces post-infarction failure, resulting in a better quality of life for patients and reduced health costs on the long term (Rajan et al., 2015). However, the main limitation of this treatment is the availability of short therapeutic window between 3 h and 4.5 h post-stroke, also due to the stringent inclusion and exclusion criteria very few patients have benefited. Only between 20% and 25% of patients arrive on time to a hospital, and between 3% and 5% of them are candidates for thrombolysis, out of which approximately 50% are reperfused (Auriel and Bornstein, 2010). Due to the complexity of cerebrovascular disease, no effective therapies for neuroprotection after acute window are available, that could prevent or block or to reverse the progressive degeneration on long term, as cognitive impairment and dementia. Therefore, new therapeutical approaches are necessaries to find effective and safety neuroprotective agents. In this review article, we highlight gene therapy for a specific gene intervention, and natural products and their derivatives as promising candidates.

## Preclinical Studies and Molecular Targets

For the last two decades, neuroprotective agents designed to block death cascade have been investigated in animal models. Numerous agents have been proposed to neurodegeneration in rodent, rabbit and primate models (Yuede et al., 2007; Van Dam and De Deyn, 2011). However, trying to solve difficulties from different kind of risk factors in VCID, as age, diabetes, hypertension, metabolic syndrome and some specific models for VCID has been proposed (Helman and Murphy, 2016; Madigan et al., 2016). Although there exist a big variability between them (Venkat et al., 2015), and the most common affection is the cognitive impairment. Therefore, an “ideal VCID animal model” is not possible, because of the existence of a wide spectrum in the definition and variation in animal models of VCID. But each preclinical animal model under controlled variable based in specific human VCI pathogenesis could give light for intervention (Jiwa et al., 2010; Helman and Murphy, 2016). Also, considering that there exist a primary or secondary convergence in the hippocampus alteration, the question could be focused in to analyze the *in vitro* and *in vivo* vascular implications.

On the other side, diverse kind of targets could be good options for blocking the progression of brain impairment and dementia (Anand et al., 2012), but this raises difficult questions like; How to define the stem event, Temporal sequence of the neurodegenerative processes, Sex and age dependent effects, when and how to carry out the intervention as well as the best analog situation in humans for a better safety and efficacy properties?

Several research groups in SouthAmerica are working in order to answer some of these questions, for example in Brazil and Argentina. Further Chile is working on methods to better understanding of neurodegeneration and looking for new treatment strategies and biomarkers for early detection of AD (Rojo et al., 2010; Beauquis et al., 2014; Vargas et al., 2014; Martino Adami et al., 2015; Nunes et al., 2015) and VaD (Valério Romanini et al., 2013; Schiavon et al., 2014; Silva et al., 2015; Primo et al., 2016). In Colombia, some experimental designs have been developed on ischemic stroke and Alzheimer’s animal models trying to propose therapeutical approach that would be versatile in the time (prevention, blocking progress and reversion) as mentioned later.

For AD studies, triple-transgenic AD mice model (3 × Tg-AD) is being used. The 3 × Tg-AD model harbors the PS1 (M146V), APP (Swe), and tau (P301L) transgenes (Oddo et al., 2003). The 3 × Tg-AD mice progressively develops Aβ plaques (3–6 months) and NFTs (12 months). In addition, 3 × Tg-AD exhibits synaptic dysfunction, including LTP deficits, like those present in an age-related manner. These deficits in long-term synaptic plasticity correlate with the accumulation of intraneuronal βA and NTFs formation. Interestingly, silenced BACE1 (main enzyme involved in the production of βA), in the hippocampus using adenoassociated viral vectors reduces the hyperphosphorilation of tau (Piedrahita et al., 2016) and improve cognitive function at 6 months and 12 months post-treatment, presenting a balanced phospholipid composition, by the reduction of saturated fatty acid (stearic acid (18:0), palmitic acid (16:0)) and increase of poli-unsaturated fatty acid (docosahexaenoic acid, DHA (22:6)), which prevented the activation of pro-inflammatory signaling cPLA2/AA/COX2 in old AD mice to long-term post-therapy (Villamil-Ortiz et al., 2016). Also, the silencing of CDK5 in the CA1 hippocampal area prevents the spreading of excitoxicity to other areas of the neuronal circuit (Piedrahita et al., 2010; Castro-Alvarez et al., 2014, 2015; Posada-Duque et al., 2015a) also reversing (Piedrahita et al., 2010) or preventing neurodegeneration by reduction of paired helicoidal formations (Castro-Alvarez et al., 2014) and further, decreases the β amyloidosis production (Castro-Alvarez et al., 2015). The result could be assumed as it involves the prevention of Calpain activation by reduction of the active p25/CDK5 complex formation (Posada-Duque et al., 2015a) with a clear impact on the down-regulation of phosphorilation rate of tau (Castro-Alvarez et al., 2015), as well as regulation of phosphatases and GSK3β activity (Castro-Alvarez et al., 2015). All of this, would lead to the control of both histopathological hallmarks, improving the neurotransmission and synaptic remodeling (Posada-Duque et al., 2016); at the same time recovering the complex synaptic molecular adhesion (p120 ctn/PSD95), which has a consequential long-term effect (12 months) in protection and improvement of cognitive function (Castro-Alvarez et al., 2015; Uribe-Arias et al., 2016). Therefore, safe and effective studies suggest that the BACE1 and CDK5 RNAi-based therapy would be ready for a potential translational trial in a patient with advanced or mild stage of Alzheimer disease, without additional option for a curative treatment.

On the other side, we take global and focal models of cerebral ischemia. In the global model occlusion of two vessels analog to a cardiac arrest is used, which develops hyperphosphorilated tau and cognitive impairment (Gutiérrez-Vargas et al., 2010; Castro-Alvarez et al., 2011). In the case of focal ischemia, the occlusion of the middle cerebral artery (t-MCAO), is useful for determining the infarct volume and the ischemic/reperfusion stroke analog to human ischemic event. There is prevelance of neurodegenerative hallmarks such as hyperphosphorilated tau, microgliosis and cognitive impairment (Gutiérrez-Vargas et al., 2015b, 2016).

Other previous research has been focused on the proposal of therapeutical alternatives with atorvastatin after the first 6 h (Céspedes-Rubio et al., 2010) and to long term post-cerebral infarction (1 month; Gutiérrez-Vargas et al., 2014), this remains valid and is potentially transferable to humans without hemorrhagic risk. As an alternative to reduceing neurological sequelae to a short and long term post-ischemia (Gutiérrez-Vargas et al., 2015a). However, CDK5 silencing in the hippocampus of ischemic rats has very impacting benefits, because it reverses learning and relearning impairment caused by cerebral ischemia, correlated with the prevention of neuronal loss, decreased hyperphosphorylated tau, reactive astroglia and microglia, within a month of post-ischemia/reperfusion (Gutiérrez-Vargas et al., 2015b). CDK5 silencing effects remain at 4 months post-ischemic, avoiding cognitive disturbance and reversing neuropathological markers, showing a potentially versatile use out of the current approved therapeutical window or in any advance stage of the VCI post-ischemic stroke. CDK5 RNAi-induced neurotransmission was a BDNF (brain derivate neutrophic factor) dependent (Gutiérrez-Vargas et al., 2016). Additional advantage shows that the targeting of CDK5 in astrocytes has protective benefits, because CDK5 RNAi induced a morphological-functional that changes in the branching and BDNF release from astrocytes protecting co-cultured neurons (Posada-Duque et al., 2015b) and increases the endotelial adhesion in ischemic brain of rats (Becerra-Calixto and Cardona-Gómez, 2016). In general, these findings suggesting that CDK5 based therapy recover the neurovascular unit integrity post-stroke (Posada-Duque et al., 2014) and also, those findings support the potential in translational studies in demented post-ischemic patients as well.

Nevertheless, looking for other translational alternatives, pharmacological therapy using natural products has a big potential. Natural products have been widely used as antioxidants or antiradicals agents, so that there are reports of inverse relationship between a diet with foods rich in antioxidants and the incidence of diseases (Sies, 1993). However, synthetic antioxidants in the food industry have proven to be responsible for liver damage and carcinogenesis and are less effective. For this reason, the use of natural antioxidants investigation has increased recently (Krishnaiah et al., 2010; Mecocci and Polidori, 2012). Natural products have a wide variety of biological effects: anti-inflammatory, anticancer, antiviral, anti-thrombotic and among others (Lee et al., 2011; Yang et al., 2014). In CNS diseases natural products have anti-convulsant antioxidant, analgesic, anxiolytic, anti-depressant, being effective in various pathologies. In addition, natural products are considered as a source of potential molecules in the field of neuroprotection (Lara-Guzman et al., 2012; Ho et al., 2013; Sumi et al., 2013; Dong et al., 2014). Therefore, in order to find pharmacological alternatives for a more agile translation to humans, a systemic administration during 3 months in a late stage of the AD mice model has been evaluated, using various natural products as flavonoids by intraperitoneal administration and monoterpenes by oral administration, which surpresively reduced the β-amyloidosis, tauopathy, cognitive disorder and anxiety present in old triple transgenic mice model of AD (Sabogal-Guáqueta et al., 2015, 2016), which could be useful in different kind of neurodegenerative environment and different advanced stages with proinflammatory markers and synaptic deterioration as VCID. Bioavailability analysis and scaling the production will increase the possibility to develop a clinical trial from South America. In summary, those experimental evidences using gene and pharmacological therapies have suggested that the neuroprotective capacity of different therapeutical strategies, convergent in blocking the change of the membrane phospholipid composition avoids the spreading of excitatory wave phenomenon and/or stopps the pro-inflammatory response in the time (Figure 1B).

## Sensor of Neurodegeneration: A Convergent Cell Phenomena in Cognitive Impairment and Dementia

Many targets are viable as strategy for preventing neurodegeneration, however based upon several evidences; the hyperphosphorilation of tau is a key phenomena that reflect cell imbalance associated to progressive brain impairment. Especially in cognitive disorder and dementia by diferent ethiologies. Tauopathy is a sensor of excitotoxicity, loss of homoeostasis, metabolic disbalance, loss of kinases/phosphateses regulation, consequences of pro-inflammatory environment, inductor of plasticity and synaptic remodeling failure are the main hallmarks of cognitive impairment and dementia (Figure 1).

AD and other tauopathies are progressive supranuclear palsy (PSP), linked to chromosome 17 Parkinson, frontotemporal dementia and cerebral ischemia, are characterized by a phenomenon of hyperphosphorylation of tau protein, causing changes in the microtubule (MT) assemblying-disassemblying dynamics (Avila et al., 2004). Tau is dissociated from MTs under the abnormal action of kinases and cytosolic accumulated as paired helical filaments (PHFs) can be bound forming NTFs (Grundke-Iqbal et al., 1986); However the condition that facilitate aggregation and formation of those structures are still unknown, stressing the importance for deeper understanding of the cellular contexts involved in such phenomenon.

Currently, 45 phosphorylation sites of tau in the brain of AD have been identified along with its 441 amino acids—that are involved in the formation of PHFs (Augustinack et al., 2002; Hanger et al., 2009). *In vitro* experiments have shown that tau is a substrate for multiple kinases including PKA, CaMKII, PKC, MAPK, MARK, CDK5 and GSK3. Among these kinases, CDK5 and GSK3 (along with their activators p35, p39, p25 and p29), has been shown to phosphorylate tau in AD related epitopes (Kobayashi et al., 1993; Paudel et al., 1993; Patrick et al., 1999). Also, the hyperphosphorilation of tau is increased by transient focal cerebral ischemia (Céspedes et al., 2013) and overall, causes impaired spatial memory (Castro-Alvarez et al., 2011), triggering in this process alteration of proteins that regulate MT assemblying. Such as CDK5, GSK3 and actin cytoskeleton, as RhoA (Céspedes-Rubio et al., 2010) and Rac (Gutiérrez-Vargas et al., 2010), small GTPases proteins and phosphatases as well. Damage of the neuronal cytoskeleton can be considered as the main cause of the loss of protein transport and neuronal instability in cerebral ischemia. MT disassemblying occurs after ischemia and plays an important role in the pathophysiology of this neurovascular disease (Pettigrew et al., 1996). Alterations in the cytoskeleton partly reflect degradation and protein aggregation after ischemia (Kühn et al., 2005). Degradation and aggregation of MAP2 and Tau hyperphosphorylation are biomarkers for the progression of ischemic damage (Pettigrew et al., 1996), and are supported by some studies showing immunoreactivity of tau in neurons and glia from thalamus, hippocampus and cerebral cortex in brain slices from people who suffered an ischemic event (Uchihara et al., 1995, 2000; Irving et al., 1996) and also in experimental cerebral ischemia models (Geddes et al., 1994; Dewar and Dawson, 1995; Irving et al., 1996).

In general, recent studies suggest that the brain pathophysiology of different kinds of VCID has a common commitment of the kinases/phosphatases balance and neurovascular integrity (Posada-Duque et al., 2014). And as a potential marker of the tissue disequilibrium progression and as a consequence of the chronic membrane composition and cell signaling alteration, inducing cognitive impairment (Llorens et al., 2016; Viswanathan et al., 2016), could be the taupathy (Figure 1).

## Human Preclinical Studies

Other strategic approach could be based in the casuistic of pure dementias in the Latin-American region, which give an exceptional opportunity to understand the pathogenesis in these human populations with genetic risk, and go in favor of the basic and clinical researchers interaction for a better understanding and medical care of mix dementias, which have more complex factors than pure ones.

The pure dementias are very rare. Most patients with dementia have mixed factors leading to dementia. Therefore, It is very common to notice AD patients with brain atrophy having vascular changes characterized by cortical infarcts, subcortical or cortico-suborticales and lecucoencefalopatía or signs of vascular suffering of the white substance which correlates with mixed dementias. Similarly, patients with VaD, also present atrophy and neurodegenerative changes that are also difficult to identify as pure case of dementia. Alzheimer’s disease and a pure case of vascular dementia.

Therefore, it is important to note that autosomal dominant AD is a pure Mendelian, neurodegenerative dementia and the autosomal dominant VaD by CADASIL is a pure VaD, the rest are vascular changes. The Mendelian autosomal dominant forms of AD and CADASIL model are two ends of a single chain. One is a model of neurodegeneration, while the other is a model of vascular disease. The comparison between these two types of dementias in clinical, neuro and pathophysiological levels will allow us to differentiate a vascular vs. a neurodegenerative dementia. But most importantly, these two models of dementia allows genetic preclinical studies providing the possibility to identify the healthy relatives carrying out the mutation in presenilin or NOTCH3 respectively and is also ideal for evaluating preventive therapies. But the more exceptional point is that these affected populations with genetic AD and CADASIL in Latin America come from large families with high average number of children per family, allowing prevention studies in population with those genetic risks.

On the other side, the search of AD treatments have evolved in the recent years. Therapeutical strategies have changed from palliative and symptomatic approach toward preventive therapies, which are considered of three types: (a) tertiary prevention therapies, consisting of treating affected population with MCI for preventing its conversion to dementia. Therefore, tertiary prevention is not properly a preclinical therapy, but it is an early intervention of the clinical phase of the disease. (b) Secondary prevention studies are aimed to prevent the onset of symptoms of AD in people who already have the disease’s neuropathological changes. Currently, few studies of secondary prevention therapies are being conducted, such as: Dominantly Inherited Alzheimer’s Network Trials Unit (DIAN-TU), testing Gantenerumab, an aggregated anti-βA antibody, and A4 (Anti-Amyloid treatment in Asymptomatic Alzheimer’s) trial, using Solanezumab (a soluble anti-βA antibody); which invite to clinically normal individuals of 65–85 years old with elevated fibrillar amyloid in brain detected by PET Amyloid to be treated (Doody et al., 2014), each drug compared to the pooled placebo. API COLOMBIA, Alzheimer’s Prevention Initiative (API-Colombia) in Latin America, which is evaluating the passive β-amyloid (βA) immunization therapy Crenezumab in cognitively unaffected persons 30–60 years old from the world’s largest known ADAD kindred in Antioquia, Colombia (Reiman et al., 2011); and API APOE4 Trial in USA will evaluate an immunization therapy in 60–75 year-old cognitively unimpaired APOE4 homozygotes. And finally, in studies of primary prevention (c), the aim is to prevent the onset of neuropathological changes of the people with high risk. However, nobody is conducting studies of primary prevention for AD or other kind of dementia. Therefore, the challenge for future is to design and to develop primary prevention studies in people with high risk for developing the disease, without symptoms or neuropathological changes. Here exist a big opportunity for developing this kind of studies, because it has been identified that there are more than 400 young carriers in the E280A FAD population.

## Translational Perspective in South America: Challenges and Pitfalls

Despite having identified potential molecular targets, there are considerable limitations inherent to the nervous system, such as crossing the blood-brain barrier and the difficulty for targeting specific neuronal populations. The presence of these obstacles has prompted the search for new strategies for the treatment of neurodegenerative diseases, using viral vectors, design of nanoparticle for improving delivery, and future approaches by systemic administration vectors or functionalized nutrition, but including safety and efficacy of the treatment remains a major challenge.

In addition to those inherent difficulties of the central nervous system, general translation of drug discovery to clinical usefulness needs to overcome several limitations, such as sensibility, specificity, well stratified patients, correct analytical approaches and differentiation from others (Drucker and Krapfenbauer, 2013). However, to promove the translation of therapeutical alternatives impacting morbility and mortality is an urgent necessity around the world and mainly in South American countries. More specifically in Colombia, there is a need to formulate policies and procedures on the subject in addition to developing a quality emergency for medical care for acute injuries (Razzak and Kellermann, 2002). Also, we need to catch up in normatives and protocols for developing clinical trials with original candidates or strategies proposed from our South American countries; and a concerted effort from scientists, medical specialists, pharmaceutical industry and government, accompanied by economical and social support. We should be able to offer experimental treatments, without false expectations to the patient/family, based in the rigorousness of the scientific preclinical evidences, a close and direct dialog with the medical team, peer-review vigilance of the I, II and III phases, and strict long-term monitoring of the potential unwanted side-effects (Main et al., 2014), are also essential. This would progressively strengthen the contribution for solving the increased mental health problems.

Now, focused in the pathogenesis, one of the most difficult aspect for developing a new strategy to block or to prevent neurodegeneration is to define a therapeutical window and its versatility to similar disorders such as VCID. For example, AD is a progressive disorder apparently preceded by a dormant period of several years, sometimes even decades before cognitive decline is evident. This normally makes it difficult to establish a suitable time window for initiating treatment. But after ischemic stroke there exist a high probability to develop cognitive impairment within weeks or months (Snyder et al., 2015; Kalaria et al., 2016), but there are no available preventive therapy. Interestingly, both neurological disorders present common phenomena like cognitive impairment and dementia that could be blocked using similar strategies. The challenge is when to intervent in each type of neurodegenerative process? (Figure 1C). Some experimental studies comparing tauopathy in Alzheimer’s animal model and ischemic stroke in rats, show that in both models exist an intrinsic homeostasis in an early period itself. But, due to loss of time this intrinsic balance is lost (Villamil-Ortiz and Cardona-Gómez, 2015; Madigan et al., 2016), generating intermediate and late period of degenerative progress that reflect in hyperphosphorylation of tau leading to loss of connectivity, therefore, this could be the common phenomenon to attack in any potential moment of the triggered cascade (Figure 1C).

In Colombia, the development of clinical trials with our own discovered therapeutical candidates have not been developed yet. A primary prevention therapy would impact the affected population and would be a boost for solving related diseases, for example like in young carriers of AD or CADASIL mutation. However, trying to put together the different and existing barriers, these are necessary: (a) discuss the inclusion and exclusion criteria of the translational medicine by interdisciplinary team of a project or institution; (b) it is necessary to prepare and to present a clinical trial protocol to a national regulatory entity, for its approval, seeking particular adjustment in each Latin American country; (c) to convince an investment agency of the need to support a clinical trial with support from original proposal from developing countries; (d) proceed step by step in the advancement of the clinical phase to the benefit as many people as possible. Finally, if this could be achieved, then it would result in providing a boost to the scientific and biotechnology development of the region.

## Conclusion

Society is being more exposed to dangerous environment and habits that induces chronic diseases, physical and mental disabilities. A Stronger Public Health normatives and social programs for prevention of unhealthy lifestyle must be developed. Non transmisible disorder triggering cognitive impairment and dementia and existing rigorous preclinical evidences that block or reverse the brain pathology disorders as presented above either by using silencing gene therapy or by systemic treatments with natural products should be further studied and researched. Colombia and South American countries should invest and support clinical trials with the understanding of the casuistic of pure dementias in patients for developing primary prevention methods. But at the same time should moderate it by normative and adequate vigilance from peer-reviewers for long-term monitoring of the potential unwanted side-effects. This action would be a great strategy for the scientific and biotechnology development of the region, and a worthy contribution to solve a major health problem.

## Author Contributions

GPC-G and FL discussed, wrote and accepted the final version of the manuscript.

## Conflict of Interest Statement

The authors declare that the research was conducted in the absence of any commercial or financial relationships that could be construed as a potential conflict of interest.
